# Peripheral Nerve Stimulator for Chronic Pain From Quadriceps Tendon Rupture: A Case Report

**DOI:** 10.7759/cureus.39916

**Published:** 2023-06-03

**Authors:** Akshat Gargya, Sampreet Dhaliwal, Naeem Haider

**Affiliations:** 1 Anesthesia and Pain Medicine, University of Vermont, South Burlington, USA; 2 Anesthesia, Trinity Health Oakland/Wayne State University, Oakland, USA

**Keywords:** chronic pain, peripheral nerve stimulation, ultrasound guided procedures, neuromodulation, quadriceps tendon rupture

## Abstract

Chronic pain from quadriceps tendon rupture (QTR) presents a significant challenge for both orthopedic surgeons and pain management physicians. Current treatment options include physical therapy and medication management. Patients with refractory pain often end up using opioids and suffer from a prolonged disability that affects the quality of their life. A peripheral nerve stimulator is a novel treatment option for QTR. It is a minimally invasive treatment option that can be used to manage refractory cases in the future. We report a case of successful management of chronic pain in a patient with bilateral QTR with a femoral peripheral nerve stimulator.

## Introduction

Due to limited evidence and consensus guidelines, chronic pain from quadriceps tendon rupture (QTR) continues to present challenges to both orthopedic surgeons and pain physicians [1]. This relatively uncommon injury has an incidence of 1.37/100,000 and can result from direct or indirect trauma, spontaneous rupture secondary to steroid abuse, chronic renal failure, gout, and rheumatoid arthritis [1-3]. Patients with QTR present with extensor lag and commonly a suprapatellar soft tissue defect on physical examination [4]. Prompt surgical repair is recommended to avoid short- and long-term complications. However, no single surgical treatment option guarantees a decreased risk of re-rupture, muscle atrophy, and chronic pain. Due to these reasons, there is a continued need for research in this patient population to find effective long-term treatment options. 

Peripheral nerve stimulation (PNS) is currently being employed for patients suffering from peripheral neuropathy, complex regional pain syndrome, acute post-surgical knee arthroplasty, ulnar neuropathy after transposition, and trigeminal/occipital neuralgia [5-11]. In the following case report, we will describe the use of femoral PNS in a patient suffering from chronic pain and opioid use due to bilateral QTR.

## Case presentation

A 64-year-old female presented to the clinic for evaluation of pain located bilaterally at the anterior and medial aspects of the thigh and knee. The pain started three years earlier when she sustained a ground-level fall directly onto her flexed knees while playing tennis. She immediately was unable to bear weight and extend her knees. She previously was prescribed opioid pain medications for unrelated chronic upper extremity pain secondary from forearm lacerations and at the time was using acetaminophen-codeine 300-30 mg every four to six hours and tramadol 50 mg tablets as needed. The patient also had a history of trigeminal neuralgia and was using gabapentin 300 mg three times daily.

She was then taken to the emergency department and initial radiographs demonstrated a large suprapatellar joint effusion on the left knee and a cluster of new calcifications in the region of the distal quadriceps tendon on the right side. The findings along with physical examination suggested avulsion fracture and distal quadriceps tendon injury. She was taken to the operating room and found to have a near 180 circumferential rent in the retinaculum/extensor mechanism with retraction of the tendon and bony fragments avulsed from the proximal patella. She underwent uncomplicated bilateral quadriceps tendon repair and post-operatively was made weight-bearing as tolerated in knee immobilizers. She underwent extensive acute inpatient rehabilitation and physical therapy post-discharge but continued to experience pain and limitation of activity.

At the time of presentation to the pain clinic, the patient described her bilateral pain as sharp, non-radiating, and 6/10 bilaterally at rest on the numeric rating scale (NRS). The pain increased to 9/10 with activity, especially when walking and climbing stairs. She was able to walk one block and could stand for about one minute before being limited by pain. The patient was unable to participate in recreational activities, especially biking, and had difficulty performing home exercises and physical therapy. She also had pain with flexion and extension of the right knee. The patient had been seen at a different pain clinic and had a femoral nerve block and adductor canal blocks three months apart. These nerve blocks provided a significant decrease in her pain which lasted only one to two months. She was using oxycodone immediate release tablet 5-20 mg q3h prn, methocarbamol tablet 500-1,000 mg q6h prn, acetaminophen tablet 1,000 mg q6h, Ibuprofen 200 mg tablet as needed, in addition to intermittent marijuana, 5% lidocaine patch, and 1% diclofenac sodium gel topically. She had also used ketamine 10%, baclofen 2%, bupivacaine 1%, cyclobenzaprine 2%, gabapentin 6%, and orphenadrine 5% compound cream with minimal benefit.

On physical examination, she had tenderness bilaterally at the anterior and medial patellar border and the popliteal angle was 20 degrees bilaterally. Flexion was 110 degrees and 90 degrees in the right and left knees, respectively, with no extensor lag bilaterally. She had a negative McMurray test and no instability to varus/valgus stress and a negative anterior and posterior drawer. The intact sensation was noted along with normal symmetric strength. A recent MRI showed post-surgical changes related to the distal quadriceps tendon repair and no evidence of significant retear or retraction of the tendon.

**Figure 1:**
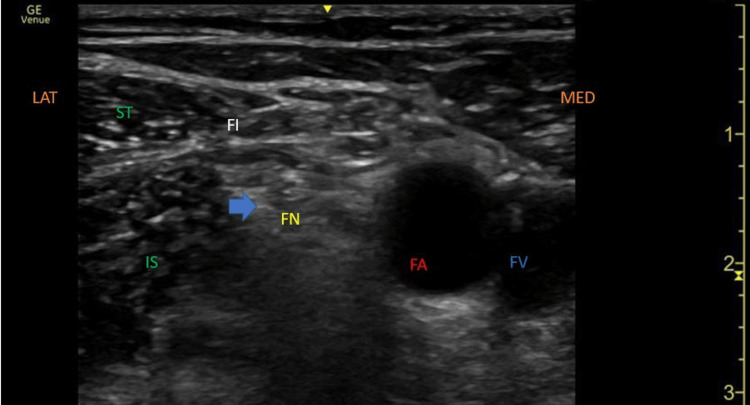
Ultrasound image using a linear array transducer demonstrating the anatomy of the femoral triangle with final lead placement FA, femoral artery; FV, femoral vein; FN, femoral nerve; IS, Iliopsoas muscle; ST, sartorius muscle; FI, fascia lliaca; arrow, final stimulating lead position; MED, medial; LAT, lateral

The patient underwent uncomplicated bilateral femoral nerve PNS placement. The technique was based on research by Singh et al. [12]. Percutaneous PNS leads were implanted 0.5 cm from the nerve to enable selective activation of large-diameter sensory fibers, under ultrasound guidance targeting the femoral nerve using a lateral in-plane approach (Figure 1).

Post-procedurally, the patient reported a significant decrease in pain in both extremities. Her pain at two months was 2/10 bilaterally. She had a painless knee range of motion of 0-130 degrees. She was able to continue her physical therapy and home exercise regimen more effectively. Her subjective physical strength had improved, and she was able to do more than 40 minutes on a lifecycle regularly. PNS leads were removed at two months and the patient reported no complications or adverse events. She was seen again at one year and she continued to have sustained pain relief with 2/10 pain in her right lower extremity and 4-5/10 pain in the left lower extremity with a continued ability to perform her tasks of daily living normally. She was able to wean off opioid pain medications and was using Tylenol 500-1000 mg q6h as needed for pain control, in addition to duloxetine 60 mg once daily. PNS hence was able to reduce pain, improve the patient's functionality, and also led to a reduction in medication use in this patient. 

## Discussion

Currently for the management of chronic QTR pain, in addition to physical therapy, commonly used therapeutic regimens include analgesic medications such as non-steroidal anti-inflammatory drugs (NSAIDs), acetaminophen, and, in rare refractory patients, opioids. Regarding physical therapy, early isometric quadriceps and hamstring strengthening exercises are recommended and active extension is generally started at six weeks. Braces and crutches are discontinued typically around 12 weeks when a patient can demonstrate sufficient quadriceps strength [13].

Like all medications, pain medications when taken chronically can cause long-term side effects including gastric ulcers, chronic kidney problems, concern for tolerance to opioids, and eventual dependence after long-term usage [14]. In a one-year prospective study, patients with a history of chronic pain or prior prescription opioid use were more likely to consume higher amounts of opioid analgesics after undergoing a surgical procedure [15]. In patients with a history of chronic opioid use and reliance on opioids, a robust patient-centric approach, and post-operative pain management plan are recommended to balance perioperative pain while simultaneously mitigating the risk of increasing opioid dosages at discharge [15]. In our patient, there was a history of both chronic pain before the QTR surgery and prior opioid use, thus making the patient susceptible to higher opioid requirements post-operatively. Thus, PNS served as a new approach to provide adequate pain control while limiting the risk of further opioid reliance.

Recent studies have demonstrated the effectiveness of PNS with a modulating effect on the central nervous system involving cortical and subcortical areas in addition to the autonomic nervous system and inflammatory pathways as a mechanism of pain relief [16-18]. The neuroinflammatory markers are modulated by PNS. Due to simulation, there is a reported increase in anti-inflammatory cytokines and a decrease in pro-inflammatory markers, especially IL-1B, and IL-6, IL-1β [16,19]. In another study, neuromodulation was shown to promote cortical activation on areas involved in pain and emotion especially by activating ipsilateral dorsolateral prefrontal and contralateral sensorimotor cortical areas during stimulation in addition to changes in regional cerebral blood flow in central pain-related regions [16,18]. In our case, we believe the patient’s pain got reduced due to its effects on central plasticity and inflammatory pathways.

PNS is minimally invasive and can be performed on an outpatient basis. Limitations of using PNS are rare. However, there is always a potential risk of bleeding, infection, incomplete coverage, and lead breakage. 2022 consensus-based guidelines described specific nerve targets that can be viable for PNS treatment [20]. It specifically explained the utility of PNS for the suprascapular, axillary, sciatic, tibial, and medial lumbar nerve branches. PNS of the femoral nerve has been described in the literature and in conjunction with the sciatic nerve can be used for post-total knee arthroplasty pain management [6,20]. Since the majority of the chronic post-surgical QTR pain is localized in the femoral nerve distribution, our case report aims to increase the awareness of the femoral nerve being a target amenable for PNS in this patient population.

## Conclusions

In conclusion, femoral PNS can be a useful treatment option for patients with chronic pain following a QTR with subsequent surgical repair. To our knowledge, there are currently no reported cases in the literature of using PNS to manage QTR pain. Hence, our case report provides a new indication for PNS use. Future adaptations and further investigations can include the implantation of a PNS device in the perioperative setting to assist in post-operative pain control and possibly decrease the likelihood of developing chronic pain. PNS has a significant potential to assist in pain control, possibly allowing patients to return to baseline daily function without the added side effects of other pain control treatments. While further research including prospective cohort studies is recommended to establish guidelines for care, our case report does provide a starting point of research regarding femoral PNS as an alternative to traditional treatments for chronic pain in patients suffering from the QTR.
